# Criminalization and coercion: sexual encounters with police among a longitudinal cohort of women who exchange sex in Baltimore, Maryland

**DOI:** 10.1186/s12954-023-00738-5

**Published:** 2023-01-27

**Authors:** Danielle Friedman Nestadt, Kristin E. Schneider, Catherine Tomko, Susan G. Sherman

**Affiliations:** 1 Department of Health, Behavior and Society, Johns Hopkins Bloomberg School of Public Health, Baltimore, MD USA; 2 Department of Mental Health, Johns Hopkins Bloomberg School of Public Health, Baltimore, MD USA

## Abstract

**Background:**

The criminalization of sex work and drug use creates unequal power dynamics easily exploited by police. Women who exchange sex (WES) in settings around the globe have reported coerced sex and sexual assault by police, and some have reported police as paying clients. Little research has examined nuances underlying WES’s sexual interactions with police.

**Methods:**

A cohort of cisgender WES (*N* = 308) was recruited through targeted sampling in Baltimore, Maryland and completed a structured survey every 6 months for 18 months. Follow-up surveys included detailed questions about recent sexual encounters with police. In bivariate and multivariate models using generalized estimating equations to account for intra-person correlation, we examined correlates of reporting recent sex with police over time.

**Results:**

One-third reported recent sex with police at any study visit. At each time point, about 90% of women who reported sex with police reported any uniformed or non-uniformed police had paid for sex. Between 72 and 85% had been solicited for paid sex by uniformed police. Between 41 and 50% of women who reported recent sex with police indicated they had done so because they feared arrest otherwise; one-third were directly pressured for sex by police to avoid arrest or trouble. In the final adjusted model, severe food insecurity [adjusted odds ratio (aOR) = 2.05; 95% confidence interval (CI) 1.13–3.71], Black race (vs. white, non-Hispanic; aOR = 1.90; 95% CI 1.13–3.17), recent arrest (aOR = 1.51; 95% CI 1.01–2.27), nonfatal overdose (aOR = 1.94; 95% CI 1.24–3.01), and client- or non-paying intimate partner-perpetrated violence (aOR = 2.46; 95% CI 1.63–3.71) were significantly independently associated with recent sex with police.

**Conclusions:**

Sexual encounters between WES and police in Baltimore are common and often coerced to avoid arrest in a setting where both drug use and sex work are criminalized. Recent sex with police was more prevalent among WES who were racially marginalized, highly structurally vulnerable, and/or at high risk for drug overdose—and therefore subject to the dual-criminalization of sex work and drug use. This indicates deep power imbalances and their exploitation by police as the root of such sexual encounters and adds to the evidence regarding the need for decriminalization to support the health and wellbeing of WES.

## Background

Sex work is illegal or restricted in most countries, and the sale and purchase of sex are fully legislatively criminalized in the USA in all but one state, though some jurisdictions have enacted policies under which prostitution/solicitation charges are no longer prosecuted [1–3]. The criminalized nature of sex work creates complex and unequal power dynamics easily exploited by police, clients, and others in ways that are detrimental to the health and wellbeing of women who exchange sex (WES). The harms caused by criminalization are, at least in part, mediated by routine and extra-judicial policing practices, and by the substantial amount of discretion police maintain in relation to enforcement and arrest under the law. Much research has focused on police discretion, and how day-to-day policing can vary dramatically even under the same laws, particularly in relation to misdemeanors such as prostitution and drug possession [4–6]. A recent study conducted in three US states found that a majority of police respondents perceived control over their decisions about misdemeanor arrest. While study participants identified supervisors as the most influential force behind their use of discretion to not arrest people for things like drug and paraphernalia possession, individual factors, such as stigma toward people who use drugs, were also significantly associated with arrest practices; perceived attitude of the suspect was identified as a critical factor in decision-making, as well [7]. Police discretion creates a point of leverage for police—ultimately they decide if they will make an arrest or not—and increases vulnerability for WES, who may be subject to inappropriate police demands if they wish to avoid arrest.

WES are at high risk for arrest and incarceration, with risk among WES who use drugs even higher, given the dual criminalization of sex work and drug use [8–10]. In the USA, there is a high degree of overlap between street-based sex work and drug use, so this dual threat applies to a large proportion of WES. Drug use has been reported both as a common motivation for entry into sex work [11, 12] and as a coping mechanism among WES.[13] One review estimated that 35–65% of WES in the USA engaged in injection drug use [14]. Lifetime criminal justice involvement has been tied to a number of adverse health outcomes, including mental health conditions, hepatitis C infection, STIs, and other infectious diseases [15–17]; it can also lead to further marginalization, as a criminal record may interfere with the ability to get housing, secure employment, and access health and social services [18]. Among WES, recent or lifetime experience of arrest or incarceration is associated with prevalent/incident HIV infection [19–23], current/recent STI infection/symptoms [19–21, 24–27], inconsistent condom use [19, 25–29], agreeing to condomless sex for more money [25, 26], and client- and police-perpetrated sexual and physical violence [21, 25, 30–33].

Evidence from settings where sex work is criminalized suggests that it fosters an environment where work-related violence is normalized, and WES may be hesitant to report abuse because of fear of adverse police responses [26, 34–36]. Many WES, across contexts, have reported lack of assistance—and sometimes arrest or further police-perpetrated violence—after reporting violence, allowing perpetrators to act with impunity [8]. Violence at the hands of police has, in turn, been associated with client-perpetrated violence [9, 25, 37]. One potential explanation for this may be due to rushing negotiations with clients or moving away from familiar areas because of police presence and associated fear of arrest or harassment [25, 37, 38].

Sexual encounters with police have been reported by WES in a variety of contexts, ranging from paid sex, to sex that is implicitly or explicitly coerced to avoid arrest, to violent sexual assault [34–36, 39–41]. In two studies in Russia, 37–38% of the samples reported sexual coercion in the past year [9, 42], and a similar proportion of a Mexican sample reported being asked for sexual favors by police in the past 6 months, with 17% reporting police sexual abuse [43]. In another study in Russia, 5% reported recent police sexual extortion, with the prevalence more than doubled among those actively engaged in injection drug use [44]; among samples in India, 9–11% reported having sex with police to avoid arrest or other trouble in the past 6 months [25, 26]. One-quarter of a sample of cisgender WES in a prior study in Baltimore had ever been pressured by police to have sex to avoid arrest [45].

Police sexual coercion has been associated with current injection drug use, past year binge drinking, selling sex on the street, rape during sex work, STI symptoms or diagnosis [9], and agreeing to condomless sex for more money [25, 42]. Broader sexual abuse by police was associated with syringe confiscation by police in Mexico[46], and any police physical or sexual violence or coercion has been associated with homelessness, recent arrest, moving to an unfamiliar location due to police presence [47], rape by non-clients [48], and client-perpetrated violence[48].

In qualitative studies, WES have described complex and nuanced dynamics underlying sexual interactions with police, but most quantitative studies that examine sexual interactions with police have focused solely on coerced sex to avoid arrest or other trouble [8, 34, 35]. One notable exception is a previous study conducted by our team, in which we found that one-quarter of a cohort of WES in Baltimore reported ever having police as paying sex work clients and that this practice was associated with recent arrest, coerced/forced sex with police, egregious police practices, and prevalent STI infection, further highlighting the overlap between experiences with police and with clients [40].

The present study aimed to examine the prevalence of sexual interactions with police, describe the circumstances surrounding these interactions in greater depth than prior studies, and identify correlates of recent engagement in sex with police, whether paid, coerced, or otherwise.

## Methods

### Setting

In Baltimore, Maryland, where this study was set, 1831.7 per 100,000 residents were living with HIV in 2019 [49], ranking it among the US cities with the highest HIV prevalence. HIV prevalence was measured at 5.2% in a recent cohort of street-based cisgender WES in Baltimore City [50]. About 1 year prior to the start of data collection for the present study, the United States Department of Justice (DOJ) published a report on their investigation of the Baltimore Police Department (BPD). The report noted a pattern of constitutional violations by BPD, grounded in a history of a “zero tolerance” enforcement strategy that sought to reduce crime by stopping and searching people, often without cause, and arresting them for any possible charge at police officers’ discretion. Although more recent BPD policy has moved away from this strategy, many supervisors' and officers' discretionary practices continued to reflect the older approaches. An audit found that between 2010 and 2015, BPD officers made several hundred thousand stops per year, concentrated heavily in poor neighborhoods with primarily Black residents, and the DOJ investigation found constitutional violations and unfounded arrests were common. Of particular relevance to the present study, the investigation found evidence that some BPD officers had coerced people involved in sex work into sexual encounters in exchange for avoiding arrest and that the BPD’s failure to adequately investigate and address such allegations had allowed for such abuses of power to recur [51]. In the same prior cohort of cisgender WES in Baltimore mentioned above, 42% of participants reported client-perpetrated violence over the time of the study (1 year), with an incidence rate of 0.78 per person year for client violence [47]; this prevalence aligns with the range for past-year workplace violence (32–55%) found by Deering and colleagues in a global systematic review of violence against sex workers [52].

### Parent study

The present study uses data from The Enabling Mobilization, Empowerment, Risk reduction and Lasting Dignity (EMERALD) study, which was a prospective two-group, non-randomized trial to assess the efficacy of a structural community-level intervention on HIV and STI risk among WES in Baltimore. The intervention was modeled after WES community empowerment approaches in international settings [53] and was comprised of a drop-in center that provided clients identifying as women, non-binary, or any non-man gender with low-barrier services, including physical and mental health care, buprenorphine for addiction management, case management, a safe space to relax, socialize, and potentially organize, and laundry and shower facilities, as well as extensive outreach activities in the surrounding neighborhoods. The baseline sample included *N* = 385 cisgender WES who completed surveys every 6 months for 18 months. The present study includes data collected at 6, 12, and 18 months study visits. More detail about the EMERALD study has been published elsewhere [54].

### Engaging women with lived experience

While many aspects of the EMERALD study protocol have been described elsewhere [54], we elaborate below on community engagement activities that have not been previously described in depth. The study and drop-in center were developed while a prior study with an active community advisory board (CAB) made up of current and former sex workers, including street-based sex workers and members of local sex work advocacy groups, was ongoing. Members of that CAB agreed to expand their scope of work to advise on development of EMERALD as well. The CAB provided input and guidance on survey questions, how to collect contact information, and various other aspects of the study procedures. They also contributed to making the research van a safe and comfortable space for participants through input on details such as what materials to give to participants and what refreshments to offer during study visits. Surveys were piloted with other women who engaged in street-based sex work in the community, who provided extensive feedback on the wording of questions, responses, etc. Efforts were made to include people with lived experience throughout the research process. The field team responsible for recruiting and following up with participants included a sex worker, and the intervention implementation team included two full-time staff members with street-based sex work experience in Baltimore City. They helped design the intervention, develop protocols, and worked directly with drop-in center clients when the center opened.

The CAB met regularly leading up to the implementation of the study and opening of the center. Members were provided direct transportation to and from the meeting location via Uber or Lyft rides arranged and paid for by the research study. They were compensated for their time with pre-paid VISA gift cards. Once the drop-in center was up and running, monthly community meetings were held with women who utilized the center to solicit feedback about research, services, and other issues within and outside of the center. Center staff have been active in advocating for and with women who exchange sex and use drugs with neighborhood associations and other groups of residents/businesses in the surrounding areas.

### Recruitment

Similar to methods used by our team in a prior study of WES in Baltimore [55], the EMERALD sample was recruited via targeted sampling. Using a series of geospatial analyses of data such as prostitution and drug arrests and 911 calls for prostitution, the study team identified “hot spots” around Baltimore City for street-based sex work activity. The research team also conducted “windshield tours” of those areas to get a sense of the sex work and other activity that was visibly present at various times of the day. These tours were conducted discreetly in unmarked cars moving with the flow of traffic in typically busy areas. Researchers noted observations about each area and did not stop or engage with sex workers during these tours. Based on this, we generated a sampling frame of different combinations of location, day of the week, and time of day, or venue-day-time units (VDTs). To increase the likelihood of recruiting a representative sample, participants were recruited in randomly selected VDTs in 10 geographic “zones”—six in the “intervention” area geographically closest to the drop-in center and four “control” zones in other parts of the city; recruitment occurred between September 2017 and February 2019. Targeted sampling allowed for adjustments throughout this period to achieve maximal coverage relative to the sampling frame [56].

Eligibility criteria were: (1) being aged 18 or older; (2) being a cisgender woman (given the small number of transgender WES in study areas); (3) having sold or traded oral, vaginal, or anal sex “for money or things like food, drugs, or favors” to clients 3 or more times in the past 3 months; and (4) being willing to provide contact information for follow up visits. Study staff discreetly approached women in designated VDTs during all hours of the day and night to invite them to participate in a study on “women’s health” to avoid any inadvertent disclosures; anyone interested in participating was screened on the study’s mobile van, and ongoing care was taken throughout the study to ensure the research team never revealed that all participants were engaged in sex exchange at baseline. Screening questions included items that were irrelevant to eligibility to mask what made one eligible or ineligible. Engagement in sex work was not required for continued participation in follow-up surveys, which helped to further mask initial eligibility criteria. These procedures were discussed with the CAB. Those who were eligible provided written informed consent if they wished to proceed after receiving detailed information about study procedures.

### Study visits

Study visits included a 50-min audio computer-assisted self-interviewing (ACASI) survey, which provided more privacy than if surveys had been administered by interviewers. The survey included sections on demographics, sex work history, drug use, and psychosocial measures, self-administered vaginal swabs for gonorrhea and chlamydia, and an oral HIV test (for those who tested negative at their prior study visit). Prior to any engagement with participants, all study staff received extensive training in human subjects’ research and good clinical practice, harm reduction perspectives and practices, including targeted training regarding harm reduction-oriented work with people engaged in sex work, supporting survivors in research on violence and adversity, and other considerations for working with street-based women who exchange sex. Training topics were informed by the CAB. Field supervisors also received formal training on HIV testing and linkage to care from the state health department, which included a focus on cultivating empathy, cultural competency, and client-centered disclosure of results [57]. At study visits, these trained supervisors provided pre- and post-HIV test counseling, as well as test results and referrals, as needed, for HIV or other neighborhood-specific services. Study staff let participants know at the start of each study visit that they were available to talk at any point if needed, and they were prepared to make warm referrals to local mental health services in case of any re-traumatization or crisis. Staff were instructed to prioritize participant safety and comfort; participants who wanted or needed to end surveys early were still compensated for the visit. Participants received $40 for 6, 12 and 18 months visits, and at the end of each visit, study staff offered local referral guides, as well as more targeted warm referrals for services participants wanted. Staff utilized several strategies used in a prior study to enhance participant retention [58]. Study procedures were approved by the Johns Hopkins Bloomberg School of Public Health Institutional Review Board.

### Measures

#### Sex with police

Each of the follow-up surveys (6, 12, 18 months) contained a series of questions about sexual encounters with police in the past 6 months, including “How many police officers have you had sex with in the last 6 months?” Numerical responses were dichotomized (any vs. none) and comprised the study’s dependent variable. Participants who reported no sex with police were asked no further questions on this topic. Those who did report sex with police were asked how many police paid them for sex (none, some, most, or all), and how many police officers they considered regular clients (none, some, most, or all) and whether police officers generally paid less, about the same, or more than other clients (asked at six- and 12-months only). An additional item at 12 and 18-months asked about how many of the police who paid for sex solicited the participant while in uniform (none, some, most, or all). All participants who reported recent sex with police, regardless of payment, were asked how often they used a condom when having vaginal or anal sex with any police officers (always, sometimes, never) and, at 6- and 12-months, whether any police had refused to use a condom when the participant wanted to. Another series of questions included in the 6- and 12-months surveys asked more explicitly about coerced sex with police. One item asked whether, in the last 6 months, the participant had “feared that if you didn't have sex with a police officer, they would arrest you,” and another asked whether a police officer had pressured the participant to have sex with them in exchange for no arrest, trouble or hassle. Participants who answered the latter question affirmatively were then asked whether they had had sex with an officer who pressured them to have sex, and those who had were then asked, “At any of those times when they pressured you to have sex with them in the past 6 months, did they still go on to arrest you after you had sex with them?”.

#### Socio-demographics and structural vulnerability

We measured age, race/ethnicity (white, non-Hispanic vs. Black vs. other race/ethnicity), and sexual minority status (“heterosexual or straight” vs. all other reported sexual orientations) at baseline. At each time point, participants were asked where they stayed most of the time in the past 6 months, and those who selected either “streets, park, car, or abandoned building (vacant)” or “shelter” were categorized as being “literally homeless” during that time period [59]. Participants reported on frequency of going “to sleep at night hungry because there was not enough food,” and those who responded five or more days per week were categorized as experiencing severe food insecurity [60]. We also assessed recent arrest for any reason (yes/no) in the past 6 months.

#### Sex work characteristics

Participants were categorized as having entered sex work as a minor if they reported that they had first exchanged sex at an age less than 18 years. We also assessed whether participants initially engaged in sex work against their will based on the question, “How or why did you initially enter the sex trade, also known as sex work or prostitution?” Participants could select all responses that applied to them, and those who reported they were “coerced, threatened or pressured,” “misled or tricked,” or “physically forced” were categorized as having entered sex work against their will. Participants also reported on each follow-up survey whether or not they had sold or exchanged sex in the past 6 months.

#### Substance use

Surveys measured type, frequency (e.g., weekly, daily, more than daily), and route of administration (e.g., snorting, injection) of various illicit/non-prescribed drugs in the past 6 months. For the present study analysis, drug use was assessed using several variables: any vs. no heroin use, daily vs. less than daily or no heroin use, any crack-cocaine use, daily crack-cocaine use, any injection of an illicit/non-prescribed drug, and daily injection, as well as two composite drug use variables: daily use of any illicit/non-prescribed drug (excluding marijuana) and daily use of more than one type of drug. Due to high correlations between various drug use variables and the high prevalence of daily use of any drug, the final adjusted model included only daily multidrug use. We also measured number of non-fatal overdoses in the past 6 months (“overdosed to the point of passing out”) and dichotomized to any versus no overdose for analysis.

#### Interpersonal violence

Violence items were taken from an adapted version of the Revised Conflict Tactic Scale [61]. On the 6-, 12-, and 18-months surveys, all participants who reported having had paying clients in the past 6 months were asked questions about violence they had experienced at the hands of clients, and all participants, regardless of their recent sex work status, were asked questions about violence perpetrated by non-paying intimate partners. Participants who reported that they had “been hit, punched, slapped, or otherwise physically hurt by [clients/intimate partners]” or had clients/intimate partners who “threatened to use or actually used a gun, knife or other weapon against [the respondent]” in the past 6 months were categorized as having experienced recent physical violence. Those who reported that any client or intimate partner in the past 6 months had “used physical force (like hitting, holding [the respondent] down, or using a weapon) to make [them] have vaginal or anal sex when [they] didn’t want to” were categorized as having experienced recent sexual violence.

#### Study zone

Participants were categorized as “intervention” versus “control” based on their recruitment location.

### Analysis

Participants were included in analyses if they responded to the item regarding sex with police at any of the three follow-up study visits. We initially examined frequencies and means of baseline characteristics and responses to the police sex items at each visit. Using generalized estimating equations (GEE) to account for intra-person correlations over time, with binomial and logit family and link functions, respectively, and exchangeable correlations, we then explored longitudinal bivariate associations between reporting recent sex with police and the other variables described above. Because no time indicators were included in the model, results reflected cross-sectional correlations over time. All variables that were significant at the *p* < 0.10 level in bivariate models, along with race/ethnicity and age, were considered for inclusion in the multivariate model, with final covariate selection guided by model fit and prior literature, as well as the goals of maximizing parsimony and minimizing collinearity. All analyses were conducted using Stata/MP 15.1 (College Station, TX).

## Results

Follow-up surveys were completed by 251, 235, and 249 participants at 6-, 12-, and 18-months, respectively. After omitting participants who could not have completed follow-up surveys because of death, incarceration, or moving out of state from the denominator, retention rates were 73%, 70%, and 64% at each respective time point. Our longitudinal sample for the present study included 308 women, who responded to the question about recent sex with police on at least one follow-up survey, and a total of 716 visits at 6-, 12-, or 18-months. At the three follow up visits, *n* = 8, *n* = 1, and *n* = 4 participants, respectively, were missing data on this item; all but two of these participants responded to the item on at least one other survey. Among the *n* = 308 participants included in analysis, 55.2% contributed data at three time points, 26.6% contributed data at two, and 18.2% contributed data at one. Participants who were homeless, injected drugs or used crack cocaine in the past 6 months, or who reported more severe depressive and post-traumatic stress symptomatology at baseline were more likely to drop out.

Baseline sample characteristics for that sample are presented in Table 1. Median age was 37 years, 56% were white, non-Hispanic, and 35% reported a sexual minority identity. Nearly one-third had been literally homeless for most of the past 6 months, and 23% had experienced severe food insecurity during the same period. Recent arrest was reported by more than a quarter of the sample (26%). Nearly a quarter (23%) of participants began engaging in sex work before the age of 18 years, and 9% reported first engaging in sex work against their will. Any and daily use of heroin and crack in the past 6 months were reported by the majority of participants, with more than half (56%) reporting any injection and just under half (46%) reporting daily injection. The vast majority (82%) of participants reported daily use of any illicit/non-prescribed drug, and 58% reported daily use of more than one drug. Recent non-fatal overdose was reported by 25% of the sample. Client- or nonpaying intimate partner-perpetrated sexual or physical violence in the past 6 months was reported by 41%.

**Table 1:** Baseline characteristics of sample, stratified by recent police sex at any visit (*n* = 308)

	Total	Police sex reported at any visit		*p*-value
		No (*n* = 217)	Yes (*n* = 91)	
*Socio-demographic characteristics*				
Age in years, median (IQR)	37 (31, 43.5)	37 (31, 44)	35 (31, 41)	0.37
Race				
White, non-Hispanic	173 (56.2%)	128 (59.0%)	45 (49.5%)	0.26
Black	114 (37.0%)	74 (34.1%)	40 (44.0%)	
Other	21 (6.8%)	15 (6.9%)	6 (6.6%)	
Sexual minority	108 (35.2%)	71 (32.9%)	37 (40.7%)	0.19
Severe food insecurity^	70 (22.8%)	42 (19.4%)	28 (31.1%)	0.025*
Mostly lived on street or in shelter^	89 (28.9%)	52 (24.0%)	37 (40.7%)	0.003*
Arrested^	81 (26.6%)	56 (26.2%)	25 (27.5%)	0.81
*Sex work characteristics*				
Entered sex work < 18 years of age	70 (22.7%)	46 (21.2%)	24 (26.4%)	0.32
Entered SW via force, coercion, or deception	29 (9.5%)	13 (6.0%)	16 (17.8%)	0.001*
*Substance use^*				
Heroin	243 (78.9%)	175 (80.6%)	68 (74.7%)	0.25
Daily heroin use	202 (65.6%)	143 (65.9%)	59 (64.8%)	0.86
Crack	261 (84.7%)	180 (82.9%)	81 (89.0%)	0.18
Daily crack use	192 (62.3%)	140 (64.5%)	52 (57.1%)	0.22
Injection drug use	172 (55.8%)	123 (56.7%)	49 (53.8%)	0.65
Daily injection	141 (45.8%)	101 (46.5%)	40 (44.0%)	0.68
Daily illicit drug use	252 (81.8%)	178 (82.0%)	74 (81.3%)	0.88
Daily multidrug use	178 (57.8%)	125 (57.6%)	53 (58.2%)	0.92
Nonfatal overdose	76 (25.2%)	47 (22.3%)	29 (31.9%)	0.078
*Violence*				
Intimate partner- or client-perpetrated sexual or physical violence^	126 (40.9%)	73 (33.6%)	53 (58.2%)	< 0.001*
*Study design*				
Intervention study arm	181 (58.8%)	123 (56.7%)	58 (63.7%)	0.25

^Past 6 months; *p < 0.05

Twenty-nine percent of the sample reported recent sex with police at any study visit (Table 2). Among those participants, the mean number of police with whom they had had sex ranged from 2.18 at 6-months to 3.18 at 18-months. At 6-months, 64% reported considering any of the police with whom they had sex regular clients; the proportion rose to 74% at 12-months. At each study visit, about 70% reported always using condoms during sex with police; at 6 months, 32% reported a police officer had refused to use a condom during sex in the past 6 months, and 19% reported this at 12 months.

**Table 2:** Frequency and distribution of police sex items

	6 months	12 months	18 months
Sex with police, past 6 months	44 (18.1%)	36 (15.4%)	45 (18.4%)
How many police (if any), mean (SD)	2.18 (1.76)	2.94 (4.30)	3.18 (5.99)
How many considered regular clients	(*n* = 44)	(*n* = 35)	–
None	16 (36.4%)	9 (25.7%)	–
Some	12 (27.3%)	11 (31.4%)	–
Most	4 (9.1%)	4 (11.4%)	–
All	12 (27.3%)	11 (31.4%)	–
How many paid			
None	4 (9.1%)	4 (11.1%)	4 (8.9%)
Some	11 (25.0%)	6 (16.7%)	14 (31.1%)
Most	2 (4.6%)	5 (13.9%)	2 (4.4%)
All	27 (61.4%)	21 (58.3%)	25 (55.6%)
Pay compared to others	(*n* = 40)	(*n* = 32)	–
Less	1 (2.5%)	2 (6.3%)	–
About same	26 (65.0%)	20 (62.5%)	–
More	13 (32.5%)	10 (31.3%)	–
How many paying clients solicited in uniform		(*n* = 32)	(*n *= 41)
None	–	9 (28.1%)	6 (14.6%)
Some	–	6 (18.8%)	16 (39.0%)
Most	–	6 (18.8%)	4 (9.8%)
All	–	11 (34.4%)	15 (36.6%)
How often used condoms			
Always	32 (72.7%)	24 (68.6%)	31 (68.9%)
Sometimes	7 (15.9%)	8 (22.9%)	9 (20.0%)
Never	5 (11.4%)	3 (8.6%)	5 (11.1%)
Police ever refused condom	14 (31.8%)	7 (19.4%)	–
Had sex with police b/c feared arrest	18 (40.9%)	18 (50.0%)	–
Police pressured for sex to avoid arrest/trouble	15 (34.1%)	12 (33.3%)	–
Had sex with police who pressured	10 (66.7%)	9 (75.0%)	–
Police arrested anyway	7 (70.0%)	4 (44.4%)	–

At each time point, about 90% of women who reported sex with police said that at least some of the time police had paid for the sex. Among those, at both time points the question was asked, about two-thirds reported police paying about the same as other clients and one-thirds reported they paid more. On the 12- and 18-months surveys, 72% and 85% of these women, respectively, reported that they had been solicited for sex by police in uniform.

Forty-one percent of women who reported recent sex with police on the 6-months survey indicated they had done so because they feared arrest otherwise, and this proportion increased to 50% on the 12-months survey. One-third were directly pressured for sex by police to avoid arrest or trouble at both six and 12 months. At 6 months, two-thirds of those women had sex with police as a result of this pressure, among whom 70% reported they were arrested anyway. At 12 months, 75% reported sex following pressure, among whom 44% were arrested.

In bivariate models (Table 3), the following variables we examined were significantly and positively associated with recent sex with police: severe food insecurity, literal homelessness, recent sex work, entering sex work via force, coercion, or deception, recent arrest, recent and daily heroin use, recent crack use, recent and daily injection drug use, daily drug/multidrug use, nonfatal overdose, and interpersonal sexual or physical violence.

**Table 3:** Bivariate and multivariate correlates of recent police sex, GEE with binomial family and logit link functions (*n* = 308 participants over 716 study visits)

	Unadjusted		Adjusted	
	OR	95% CI	OR	95% CI
*Socio-demographic characteristics*				
Age	0.98	0.95–1.00	0.98	0.96–1.01
Race				
White, non-Hispanic	Ref		Ref	
Black	1.44	0.89–2.34	1.9*	1.13–3.17
Other	0.73	0.28–1.89	1.14	0.45–2.86
Sexual minority	1.12	0.70–1.80	–	–
Severe food insecurity^	2.9**	1.73–4.83	2.05*	1.13–3.71
Mostly lived on street or in shelter^	1.66*	1.07–2.58	1.2	0.74–1.94
Arrested^	2.23**	1.51–3.29	1.51*	1.01–2.27
*Sex work characteristics*				
Recent sex work	2.86*	1.56–5.24	1.42	0.73–2.77
Entered sex work < 18 years of age	1.19	0.70–2.00	–	–
Entered SW via force, coercion, or trickery	2.17*	1.18–3.99	–	–
*Substance use^*				
Heroin	2.1*	1.27–3.49	–	–
Daily heroin	2.36*	1.45–3.86	–	–
Crack	2.1*	1.20–3.67	–	–
Daily crack	1.51	0.99–2.30	–	–
Injection drug use	1.83*	1.21–2.76	–	–
Daily injection	2.05*	1.32–3.19	–	–
Daily illicit drug use	2.3*	1.29–4.12	–	–
Daily multidrug use	2.45**	1.54–3.92	1.53	0.87–2.70
Nonfatal overdose	2.56**	1.72–3.80	1.94*	1.24–3.01
*Violence*				
Intimate partner- or client-perpetrated sexual or physical violence^	3.62**	2.46–5.33	2.46**	1.63–3.71
*Study design*				
Intervention study arm	1.17	0.72–1.90	1.32	0.79–2.18

^Past 6 months

*p < 0.005

**p < 0.001

In the final adjusted model (Table 3), severe food insecurity [adjusted odds ratio (aOR) = 2.05; 95% confidence interval (CI) 1.13–3.71; *p* = 0.017], Black race (vs. white, non-Hispanic; aOR = 1.90; 95% CI 1.13–3.17; *p* = 0.015), recent arrest (aOR = 1.51; 95% CI 1.01–2.27; *p* = 0.045), nonfatal overdose (aOR = 1.94; 95% CI 1.24–3.01; *p* = 0.003), and client- or non-paying intimate partner-perpetrated violence (aOR = 2.46; 95% CI 1.63–3.71; *p* < 0.001) were all significantly independently associated with recent sex with police.

## Discussion

In this study, we found that nearly one-third of our sample had engaged in recent sex with police at any study visit. While we deliberately explored this broad outcome to allow for multiple types of sexual encounters and the possibility that WES might actively choose to engage in these encounters, our findings suggest that structural vulnerabilities play a significant role and may limit the degree of agency WES are able to exercise. The criminalization of sex work and drug use, in conjunction with deep social and structural stigma surrounding these behaviors, creates power imbalances, whereby police may act with impunity and WES have little recourse to address abuses of power.

The vast majority of women in our sample reported that they were paid by at least some of the police with whom they had sex, and while most of them said police paid the same amount or more than other clients, most also reported that they had been solicited by police in uniform, which may serve as a reminder of the additional power police clients hold relative to non-police clients, given their ability to decide to arrest WES at any point. This aligns with prior qualitative literature from Baltimore, in which WES reported that police most often made sexual advances while on duty, and their ability to decline or negotiate the terms of sexual encounters were limited by the ever-present threat of arrest and the status and power wielded by police [34]. In a prior cohort of WES, we also found that women who reported recent arrest, coerced or forced sex by police, or a higher number of egregious policing practices were more likely to have police as clients, highlighting the close connections between routine policing, sexual and other types of exploitation by police, and the “everyday violence” of having police as clients [40]. In the present study, at the two visits where the items were asked, about half of the sample reported either having sex with police because they feared arrest or that police had explicitly pressured them for sex to avoid arrest or trouble (combined variable not shown in tables). At the 6- and 12-months visits, respectively, 100% and 92% of women experienced implicit or explicit coercion and/or were paid. Together, prior findings about the associations between paid police sex and experiences of sexual exploitation and violence by police and the present study’s findings about the role of coercion in sex with police suggest sexual encounters with police among this sample are likely more akin to coercion or violence than to voluntary relationships.

Daily drug use was also reported by the vast majority of this sample. Studies in various settings have found that WES who use drugs are more likely to experience police encounters, arrest, incarceration, and violence, highlighting the compounding effects of dual criminalization of sex work and drug use for WES who use drugs [8–10]. In bivariate models, recent sex with police was associated with literal homelessness, Black race, severe food insecurity, daily drug use, recent overdose, recent interpersonal violence, and recent arrest. All but homelessness and daily drug use remained significant in the adjusted model, possibly because of high correlations with food insecurity and overdose, respectively. These are all markers of structural vulnerability, driven by these women’s often multiply marginalized positions in the social structure and the criminalization of sex work and drug use [62]. They are also factors that have been tied to police coercion and violence in various populations. For example, in the USA, intimate partner violence has been significantly associated with police-perpetrated sexual violence [63, 64], and the elevated risk of police violence—including sexual violence—among people of color has been well-documented [51, 63–66]. At least one New York-based study found that, among Black women in mandated community supervision programs, sexual abuse by police was associated with more than double the odds of overdose [67]. Further highlighting the impact of intersectional marginalization, a study of people who inject drugs in California found that the prevalence of sexual violence or exploitation by police increased with each additional marginalized identity held by participants—being female, reporting a history of sex work, being homeless, and being a non-white race [68]. Most studies related to police abuse among WES explore sexual violence and coercion as predictors of other outcomes rather than exploring factors that may increase vulnerability to sexual exploitation by police [8], which makes direct comparisons to studies challenging. However, WES engaged in sex work for survival—who are contending with food insecurity, housing instability, and the need to avoid drug withdrawal—may have little agency to dictate the terms of sexual encounters and occupy a particularly disadvantaged position of power relative to the police [34, 69, 70].

### Limitations

The study is subject to several limitations. Our survey did not include items about forced sex or physical violence from police, which could have provided additional important context. For example, a small proportion of women in our sample (*n* = 3; 8.3% at the 12-months visit) reported sex with police and did not report being paid or being implicitly/explicitly coerced into it to avoid arrest. It is possible that these women were engaged in voluntary, unpaid sexual relationships with police, and it is also possible that they were raped, and these scenarios have wildly different connotations. Future research in this area would benefit from a mixed methods approach to better understand these issues. In addition, because survey items regarding interactions with sex work clients more broadly did not instruct participants to exclude police clients from their responses, we are unable to directly compare the level of exploitation or abuse involved in encounters with police to those with clients holding other occupations.

The parent study utilized a community advisory board made up of people currently or formerly engaged in sex work, and members of this board and other people with lived experience were involved in various aspects of the study. However, though research team offered engagement in manuscript-writing to various of these partners with lived experience, ultimately none participated in data analysis or writing, including for the present manuscript. As a result, we may have failed to explore or capture some critical nuances related to this issue.

Although we utilized data from multiple time points, our results reflect cross-sectional associations, and, thus, we cannot draw any conclusions about temporality or causality. We also cannot assess the degree to which these findings are generalizable beyond our sample or how representative this sample is of the population of cisgender WES in Baltimore. Our use of targeted sampling aimed to recruit as representative a sample as possible, but, as the characteristics of the population are unknown, we cannot measure how successful we were. Between 2018 and 2021, 64% of sex work arrests among people categorized as female were of non-Hispanic white women, and 35% of non-Hispanic Black women [71]. This does not represent demographics of individuals who were arrested because some may have been arrested, and therefore counted, more than once. However, these numbers are similar to the racial breakdown of our sample (56% non-Hispanic white and 37% Black) adding some support to the representativeness of the sample. The study sample did not include transgender WES, and, thus, we cannot draw any conclusions about sexual interactions between that group and police, which are likely distinct, based on the literature from Baltimore and other settings [8]. Data for this study were collected prior to a 2020 decision by Baltimore City’s State’s Attorney’s Office to stop prosecuting prostitution and drug possession cases [3], which may have led to some changes in WESs’ encounters with police, including sexual interactions. Arrest data suggest that average monthly arrests for drug possession decreased by 82% in the 13 months after the policy was enacted. In that same period of time, there were no arrests for prostitution (there was an average of 25 arrests per month before the policy change) [72]. However, it is unclear to what extent that reduction has been matched by decreases in negative interactions with police. Ongoing research is exploring how policing practices and power dynamics have or have not evolved under this de facto decriminalization of sex work, and we are not aware of prior literature that has examined this.

## Conclusions

Sexual encounters between WES and police in Baltimore are common and are often coerced to avoid arrest in a setting where both drug use and sex work are criminalized. In our study, reporting sex with police was more prevalent among WES who were racially marginalized, highly structurally vulnerable, and/or at high risk for drug overdose—and therefore subject to the dual-criminalization of sex work and drug use. This indicates deep power imbalances and their exploitation by police as the root of such sexual encounters and adds to the evidence regarding the need for decriminalization to support the health and wellbeing of WES.

## Data Availability

The datasets generated and/or analyzed during the current study are not publicly available due to their containing information that could compromise the privacy of research participants but are available from the senior author, SGS (ssherman@jhu.edu), on reasonable request.

## Abbreviations

ACASI: Audio computer-assisted self-interviewing
aOR: Adjusted odds ratio
CAB: Community advisory board
EMERALD: Enabling Mobilization, Empowerment, Risk Reduction and Lasting Dignity (study name)
GEE: Generalized estimating equations
VDT: Venue-day-time unit
WES: Women who exchange sex
